# Suboptimal management of severe menopausal symptoms by Nigerian Gynaecologists: a call for mandatory continuing medical education for physicians

**DOI:** 10.1186/1472-6874-9-30

**Published:** 2009-10-23

**Authors:** Peter O Nkwo

**Affiliations:** 1 Department of Obstetrics and Gynaecology, University of Nigeria Teaching Hospital, Enugu, Nigeria

## Abstract

**Background:**

Effective management of menopause is an important way to improve the quality of life of the increasing number of older women. The study sought to find out if Nigerian Gynaecologists offer effective treatment for severe menopausal symptoms.

**Methods:**

126 Nigerian Gynaecologists representing the six health zones of Nigeria were interviewed to determine the menopausal symptoms they had ever encountered in their practices, frequency of the symptoms, treatments ever offered for severe symptoms including their attitude to, and practice of hormone replacement therapy.

**Results:**

A Nigerian Gynaecologist encountered an average of one patient with menopausal symptoms every three months (range: 0-3 patients per month). The commoner symptoms they encountered were hot flushes (88%), insomnia (75.4%), depression (58.0%), irritability (56.3%), night sweats (55.6%) and muscle pains (54.8%) while urinary symptoms (16.7%) and fracture (1.6%) were less common. Treatments ever offered for severe symptoms were reassurance (90.5%), anxiolytics (68.3%), analgesics (14.3), HRT (7.9%), Vitamins (4%), Beta-blockers (3.2%) and Danazol (2.4%). These treatments were offered as a matter of institutional traditions rather than being based on any evidence of their efficacy.

**Conclusion:**

The result revealed that most Nigerian Gynaecologists prefer reassurance and anxiolytics for managing severe menopausal symptoms instead of evidence-based effective therapies. A policy of mandatory continuing medical education for Nigerian physicians is recommended to ensure evidence-based management of gynaecological problems, including menopause.

## Background

Effective management of menopause is necessary to improve the quality of life of the growing population of menopausal women. The increasing life expectancy in most societies means that more than ever before, women are now attaining menopause and spending more years in a state of ovarian failure. The age of menopause has remained constant at about 51 ± 3 years [1-3]. With an average life expectancy of 82 years in Britain for instance, an average woman now spends about a third of her life-time in the menopausal state. Ovarian failure is associated with profound changes which have both short term and long term consequences for the woman's quality of life [4]. Relatively simple hormone treatments are effective for this condition [4-6]. Non-hormonal treatments have also been employed for many years with varying degrees of reported successes [4,7-10]. However, only hormone replacement therapy (HRT) is believed to address the root cause of the symptoms, and has proved effective for symptomatic relief as well as for prevention of the long term sequellae of menopause [4,11,12]. HRT remains the reference standard for assessing the effectiveness of non-hormonal interventions. The results of the Women's Health Initiative Survey [13] and subsequent studies [14,15], have revealed evidences of increased risks of breast cancer, stroke and pulmonary embolism in association with long term use of hormone replacement therapy. HRT is however still considered justifiable and indeed recommended for symptomatic relief of severe menopausal symptoms although it is no longer recommended for long-term prophylaxis [5,14-16]. Physicians who care for women are expected to be familiar with evidence-based effective and safe management of menopause.

In Nigeria, there is dearth of information on the subject menopause. Because few women presented to hospital with menopausal symptoms, it was assumed in the past that most Nigerian women did not develop menopausal symptoms or that the symptoms were so mild that the women could cope without medical treatment. Hence, the average Nigeria-trained physician did not consider menopausal symptoms as important health problems. Recent community-based studies [17,18] have revealed that most menopausal symptoms are indeed prevalent amongst Nigerian women. Because of cultural practices that engender positive attitude to menopause in some Nigerian communities [17], women with mild menopausal symptoms are able to cope with the symptoms without seeking for treatment. However, women with moderate and severe menopausal symptoms consider them important health concerns and seek treatment for these symptoms. According to Agwu et.al [18] women who consulted physicians for menopausal symptoms were not offered effective treatments of their symptoms whereas those who consulted the herbal medicine practitioners were offered more effective herbal therapies most of which consisted of phytoestrogen preparations. As a result, the botanical preparations are very popular among the menopausal women while hospital consultations for menopausal symptoms are unpopular. Could the paucity of hospital consultations for menopausal symptoms in Nigeria be the result of ineffective hospital-based treatment of these symptoms? The overall objective of this study was to determine whether or not Nigerian Gynaecologists offer effective and evidence-based treatment of severe menopausal symptoms. The specific objectives were to determine the menopausal symptoms ever encountered by Nigerian Gynaecologists in their practices, the relative frequencies of the symptoms, the types of treatment they had ever offered for severe menopausal symptoms and their attitude to, and practice of hormone replacement therapy. The results of the study are expected to be particularly useful to the Society of Gynaecology and Obstetrics of Nigeria (SOGON) and the Postgraduate Medical Colleges in their respective functions of regulating gynaecology practice and training of Gynaecologists in Nigeria.

## Methods

This cross sectional study was conducted amongst Nigerian Gynaecologists who were attending an annual conference. The study population was the 540 Gynaecologists in the register of the Society of Gynaecology and Obstetrics of Nigeria (SOGON) in 2006 [19]. A minimum sample size of 109, representing 20% of study population was considered an adequate representative of the study population. In order to ensure proportionate representation, the respondents were first stratified into 6 according to the 6 health zones of Nigeria. Next, 20% of the registered members of SOGON from each health zone was calculated to obtain the minimum sample size for the corresponding zone. The calculated minimum sample sizes for the 6 health zones were as follow; South-West = 35, South-South = 27, South-East = 22, North-Central = 13, North-West = 6 and North-East = 6. To increase statistical power of the study whilst maintaining the zonal proportionality of the participants, a maximum of 20% increment of the calculated minimum sample size was allowed for each zone. Hence the maximum expected sample size was 134. The expected range of participants from each zone was as follow; South-West zone = 35-42, South-South zone = 27-33, South-East = 22-27, North Central = 13-16, North-East = 6-8, North-West = 6-8. There was plan to conduct a follow-up survey in any zone that did not have its minimum sample size.

For the interview process, the purposes of the study were explained individually to consecutive attendees at the SOGON annual conference immediately following registration and they were solicited to participate in the study. Those who consented were interviewed with the aid of self-administered semi-structured questionnaire until the maximum sample size for each zone was reached. The questionnaire captured socio-demographic data, length of practice as a Gynaecologist, geographical location of practice, menopausal symptoms ever encountered by the respondents in their practices, the treatments ever offered for severe menopausal symptoms, their attitude to hormone replacement therapy, and their practice of hormone replacement therapy.

Data entry, collation and analysis were done with SPSS statistical software version 10 for descriptive statistics, Z-test and Chi-Square test as appropriate. P value of = 0.05 was considered significant (at 95% confidence interval). This study was approved by the research ethics committee of the University of Nigeria Teaching Hospital, Enugu Nigeria.

## Results

Out of the 151 eligible conference attendees interviewed, 126 returned their completed questionnaires, giving a response rate of 83%. Nigeria is divided into 6 health zones. The zonal distribution of respondents was as follow: South-West = 38, South-South = 32, South-East = 27, North-Central = 14, North-East = 8 and North-West = 7. The ages of the participants ranged from 35 years to 61 years with a mean of 47.71 ± 0.84 years. They had practiced as specialist Gynaecologists for a mean of 6.83 ± 0.54 years with a range of 1 to 34 years. On the average, a Gynaecologist saw one patient with menopausal symptoms every three months (range: 0-3 patients per month). The main menopausal symptoms they had ever encountered are shown in Table 1. The commonest symptom was hot flushes while fracture and urinary symptoms were uncommonly reported. Table 2 shows the treatments ever offered by the Gynaecologists for severe menopausal symptoms. Reassurance and anxiolytics were the common treatments while HRT was infrequently used. Attitude to HRT was assessed by asking whether they considered HRT more effective than the non-hormonal therapies for the treatment of severe menopausal symptoms. 6.3% agreed that HRT is more effective than the other treatment modalities they offered while 93.7% disagreed. The reasons for non-use of HRT are shown in Table 3. Non-availability of HRT was the reason given by 59.5% of the respondents for non-use of HRT while only 10.3% of respondents gave side effects of HRT as their reason for its non-use.

**Table 1:** Menopausal symptoms ever encountered by Gynaecologists in Nigeria (N = 126)

**Symptoms**	**Number of respondents**	**% respondents**
Hot flushes	112	88.9

Insomnia	95	75.4

Depression	73	58

Irritability	71	56.3

Night sweat	70	55.6

Muscle/joint pain	69	54.8

Vaginal dryness	60	47.6

Easy fatigability	54	42.9

Reduced libido	51	40.5

Crawling sensation	48	38.1

Hypertension	33	26.2

Urinary symptoms	21	16.7

Fracture	2	1.6

**Table 2:** Treatment ever offered by Nigerian Gynaecologists for severe menopausal symptoms (N = 126)

**Treatment**	**Number of respondents**	**% of respondents**
Psychotherapy	114	90.5

Anxiolytics	86	68.3

Analgesics	18	14.3

HRT	10	7.9

Multivitamins	5	4.0

Beta-blockers	4	3.2

Danazol	3	2.4

**Table 3:** Reasons for non-use of HRT by Nigerian Gynaecologists (N = 126)

**Reason**	**Number of respondents**	**% of respondents**
Non-availability	75	59.5

High cost	20	15.9

Side effects	13	10.3

Lack of experience	9	7.1

Patients' poor adherence	6	4.8

Fear of abuse of the drugs	3	2.4

## Discussion

The zonal distribution of participants in this study, especially the North-South disparity, is consistent with the national distribution of Gynaecologists in Nigerian [19,20] and each health zone was represented by at least 20% of its registered Gynaecologists. The study revealed that although most reported menopausal symptoms [17,18] are prevalent amongst Nigerian women, the average Nigerian Gynaecologist sees only a small number of such cases each year. Whereas hot flushes and psychosomatic symptoms are commonly reported, uro-genital symptoms are less commonly reported while fracture is rarely reported. The widespread social inhibition and secrecy about female sexual and genital matters in most Nigerian communities may be responsible for the low reporting of sexual and genital symptoms such as vaginal dryness, dyspareunia, reduced libido and urinary symptoms. Women are much less embarrassed about reporting the non-genital and non-sexual complaints such as hot flushes, crawling sensation, insomnia, irritability and depression, hence the apparent higher prevalence of these non-genital and non-sexual symptoms. Information from focused group discussions however, revealed that Nigerian menopausal women experience the urinary and genital symptoms. These symptoms are usually presented to the clergy, spiritualists and traditional medicine men rather than to the hospitals [18]. Nkwo et.al [17] recorded only one case of fracture while Agwu et.al [18] reported no case of fracture in their studies. The black woman is blessed with a large bone mass which may explain the protection of Nigerian women from menopausal fracture. On the other hand, women with menopause-induced osteoporotic fracture are more likely to be found in orthopaedic wards and homes and not in the Gynaecology clinic and church service (as in the study of Nkwo et.al) or in a village square meeting (as in the study of Agwu et.al) and so may have been unintentionally excluded by the study designs!

The common use of reassurance and anxiolytics for treating menopausal symptoms was probably influenced by the high prevalence of psychosomatic symptoms. It is however more likely that such treatments resulted from a common but wrong opinion held by many Nigerian doctors in the past that the typical Nigerian women do not manifest severe menopausal symptoms, hence reassurance evolved as the first line management of menopausal symptoms. Women who did not respond to reassurance were given analgesics and anxiolytics as well. By practice tradition, generations of Nigerian doctors learnt to treat menopausal symptoms, including severe symptoms, with reassurance, anxiolytics and analgesics. Although some societal privileges enjoyed by menopausal women in some Nigerian communities tend to modulate the expression of menopausal symptoms; they do not eliminate them [17]. Indeed, some Nigerian women are known to have presented with quite severe menopausal symptoms that are not mitigated by reassurance, anxiolytics or analgesics but by hormonal or effective non-hormonal therapies.

It is worrisome that most of the respondents (93.7% of them) did not consider HRT more effective than reassurance, anxiolytics and analgesics for severe menopausal symptoms. This revealed an important knowledge gap amongst the respondents, leading to suboptimal treatment of these symptoms. The superiority of HRT over placebos for severe menopausal symptoms is established [5,7,14,16]. Moreover, reassurance, analgesics, anxiolytics, and multivitamins are not among the documented effective non-hormonal therapies for severe menopausal symptoms [10]; their widespread use by Nigerian Gynaecologists is not based on any documented evidence. Caution in the use of HRT resulted from its potential cardiovascular and oncogenic side effects following prolonged usage [12,15]. Incidentally, only 10.3% of the respondents withheld the use of HRT for fear of its side effects.

Patient satisfaction is an important determinant of health seeking behaviour. Dissatisfaction with treatments offered by the physicians might be the major reason why only few women present to them with menopausal symptoms. Instead, the women seek treatment from alternative sources including botanical preparations some of which are quite popular among the women possibly as the result of their effectiveness in relieving the menopausal symptoms. There is an urgent need for Nigerian Gynecologists to regain the confidence of the Nigerian menopausal women by offering them effective management of their menopausal symptoms. The Society of Gynaecology and Obstetrics of Nigeria (SOGON) and the Post Graduate Medical Colleges are encouraged to take particular note of studies on practice patterns and institute mandatory continuing medical education programmes to bridge observed gaps and so encourage evidence-based best practices rather medical practice based on existing traditions.

## Conclusion

Most Nigerian Gynaecologists offered suboptimal and ineffective treatments for severe menopausal symptoms. This resulted from medical practice that is still strongly influenced by tradition rather than evidence. Continuing medical education of physicians is recommended to encourage evidence-based best practice of medicine in Nigeria.

## Competing interests

The author declares that they have no competing interests.

## Author's contributions

PN conceived the study, prepared the questionnaire, supervised the data collection, collation and analysis and wrote the manuscript.

## Pre-publication history

The pre-publication history for this paper can be accessed here:


